# Prevalence of Hypertension in Indian Tribes: A Systematic Review and Meta-Analysis of Observational Studies

**DOI:** 10.1371/journal.pone.0095896

**Published:** 2014-05-05

**Authors:** S. A. Rizwan, Rakesh Kumar, Arvind Kumar Singh, Y. S. Kusuma, Kapil Yadav, Chandrakant S. Pandav

**Affiliations:** 1 Centre for Community Medicine, All India Institute of Medical Sciences, Ansari Nagar, New Delhi, India; 2 Indian Coalition for Control of Iodine Deficiency Disorders, New Delhi, India; University of Bologna, Italy

## Abstract

**Introduction:**

In India there is an increasing trend in hypertension prevalence among the general population. Studies have shown that tribal populations in India are also experiencing this burden.

**Objective:**

The aim was to estimate the pooled prevalence of primary hypertension among adult tribal populations of India.

**Methods:**

A systematic search was conducted in MEDLINE, IndMed, Web of Science, Google Scholar and major journals for studies published between 1981 and 2011. Two authors independently reviewed the studies, did quality assessment and extracted data in pre-coded spread-sheets. Pooled estimates of prevalence of hypertension were calculated using *DerSimonian-Laird* random effects model. Subgroup and sensitivity analyses and meta-regression were performed.

**Results:**

Twenty studies or 53 subpopulations with 64 674 subjects were included in final review. The pooled estimate of hypertension prevalence was 16.1% (95% CI: 13.5, 19.2). There was significant heterogeneity among the studies (I^2^ = 99% and Q = 4624.0, df  = 53, p<0.001). Subgroup analyses showed that year of study, acculturation status, special features, and BP measurement techniques significantly influenced prevalence, but after meta-regression analyses, ‘decade of study’ remained the only covariate that significantly and independently influenced prevalence (R^2^ = 0.57, Q = 119.2, df  = 49, p value <0.001).

**Conclusion:**

An increasing trend was found in the prevalence of hypertension in adult tribal populations across three decades. Although acculturation was probably the underlying agent that caused this increase, other unmeasured factors that need further research were also important. Concerned policy makers should focus on the changing health needs of tribal communities.

## Introduction

Cardiovascular Disease (CVD), one the major causes of death in developed nations, is increasingly being recognized as a major killer in developing nations like India [1]. Although CVD has a wide gamut of risk factors, primary hypertension remains a major underpin that accelerates its risk. High blood pressure is responsible for 7% of global Disability Adjusted Life Years (DALY) loss, and by 2025 about 29% of world's populace is projected to suffer from this condition [2], [3]. Primary hypertension has particularly intrigued the scientific community because of its amenability for community level intervention and primary prevention. In hypertension research, tribal populations provide an interesting epidemiological window, since studies world over have shown that they have a lower prevalence, and that their Blood Pressure (BP) does not rise with age [4]–[11]. However, recent studies reported high prevalence among tribes in India [12]–[14]. In India, tribes constitute 8% of the total population with an overwhelmingly diverse range of types [15]. Tribal populations are less accessible for scientific study because of their scattered habitats, inaccessible terrain, and nomadic nature of living. This meta-analysis was done to estimate the prevalence of primary hypertension among adults of various tribal groups in India for the period 1981 to 2011, and to investigate possible sources of heterogeneity in the estimate.

## Methodology

### Literature search strategy

The literature search was carried out independently by two authors (RSA, RK). Disagreements on study inclusion, quality assessment, and data extraction were resolved by deliberation or by a third author (AKS). We searched databases like Medline, IndMED, Web of Science, and Google Scholar. We screened table of contents of journals which were likely to publish such studies. Websites and published documents of national agencies like National Nutrition Monitoring Bureau (NNMB), related organizations, and the Ministry of Tribal Affairs were searched. Cross references of all selected articles were scanned for additional studies. Attempts were made to retrieve grey literature like unpublished data, dissertations, and conference proceedings. To obtain disaggregate data, at least two email requests were sent to the corresponding author. If more than one article was published from a study, the article that provided the most updated data was selected. Study selection criteria are shown in box 1 and full search strategy is detailed in Boxes S1 and S2. The last date of literature search was 10^th^ October, 2012.

### Quality assessment and data extraction

Using appropriately modified critical appraisal checklists, each article was assessed for quality by two authors (Box S3) [16]. Study characteristics (first author, place of study, year of publication - representing year of study, sampling scheme, sample size, BP apparatus, number of BP readings, and classification cut-offs), participant characteristics (age group, tribe name, status of acculturation, and special features), and prevalence were extracted onto pre-coded spreadsheets independently by two authors (RSA, RK). Data were extracted at the lowest possible disaggregate level (referred to as subpopulation here). If tribe wise disaggregate data were not available, the next highest level was taken to represent a tribe (for example, village level). This review is presented according to the PRISMA/MOOSE statement. (Checklist S1, Table S1) [17].

### Statistical Analysis

Effect size of interest was proportion of individuals with hypertension. All meta-analyses were done in logit scale due to their desirable statistical properties and by using DerSimonian-Laird random effects model [18]. Final results were transformed back to proportion for interpretation. Subgroup analyses were done for sex, age, time period, region, acculturation, special features, BP recording procedures, classification cut-offs, and sampling strategy. For estimating secular trend, point estimates for three separate decades were calculated and trend line fitted. A meta-regression analysis was performed to determine the effect of covariates on prevalence by using random intercept fixed slopes analysis (maximum likelihood estimation method). Regression coefficients for logit were presented with 95% confidence intervals. Sensitivity analyses were performed by discarding low quality studies, by removing outlier subpopulations (point estimates >3 SD), or by removing smaller subpopulations (size <100). We used Comprehensive Meta-Analysis version 2.2 (Biostat, Englewood, NJ) and Stata/IC version 11.1 (StataCorp LP, College Station, TX) for analyses. Heterogeneity between studies was examined using I^2^ and Cochran's Q statistics. Publication bias was assessed by visual inspection of funnel plots and ‘Duval and Tweedie's trim and fill technique’. Statistical significance was set at p value <0.05.

## Results

**Figure 1:**
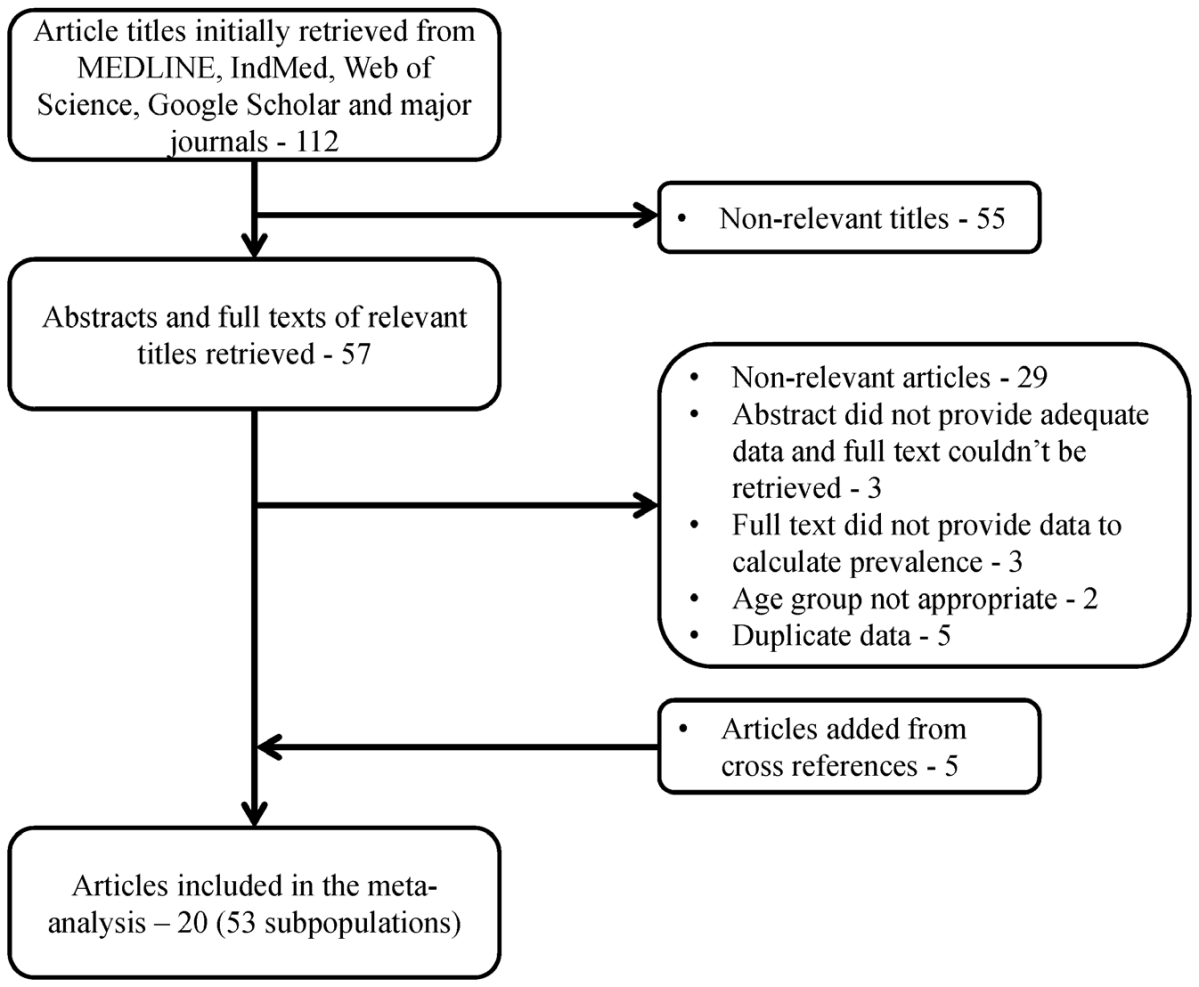
Flow of selection of studies.

The flow of article selection is shown in Figure 1. A total of 20 articles (53 subpopulations) were finally included in the review (for references see Text S1). All included studies were cross-sectional surveys. The total number of subjects included was 64 674, with 31 565 females and 27 533 males. Information regarding sex was not available for 5576 participants. Table 1 summarizes the major study characteristics. Except for one study that included a slightly older age group (≥30 years), all other studies included a uniform age group (mostly above 18 years). Quality assessment showed that 13 studies were of high quality (Table S2). Based on geographical continuity, places of study were clubbed into three regions, a) Himalayan and north-eastern, b) central, and c) southern (Figure S1). A number of tribes with varied characteristics were considered in these studies (Table S3). Studies had several methodological differences. Majority of the studies employed a random sampling strategy, used mercury sphygmomanometer, employed multiple BP recordings, and used a 140/90 mm Hg classification cut-off. Prevalence of hypertension for both sexes combined (n = 17 studies) ranged from as low as 0% in a study done by Reddy BN [19], in Andhra Pradesh in 1998 with a size of 156 to as high as 51% by the NNMB study [20], done in 2009 in Orissa with 2859 participants. The same ranged from 0% to 50% and 0% to 80% among females (n = 13 studies) and males (n = 13 studies) respectively.

**Table 1:** Characteristics of studies (subpopulations) included in the review.

Author (year)	State	Age (years)	Acculturation	Special features	Sampling scheme	BP apparatus	No of readings	Cut-off	Prevalence of HTN (males) % (No of hypertensives/total no of males)	Prevalence of HTN (females) % (No of hypertensives/total no of females)	Combined prevalence of HTN*, % (No of hypertensives/total no of participants)
Dasgupta DJ et al (1982)	Himachal Pradesh	>14	No	No	Random	Mercury	Multiple	160/95	1.2 (5/412)	2.5 (14/570)	1.9 (19/982)
Puri DS et al (1986)	Himachal Pradesh	>14	No	No	Non-random	Mercury	Multiple	160/95	NA (NA/1592)	NA (NA/1511)	2.4 (74/3103)
Dash SC et al (1994) {A}	Orissa	>19	No	No	Random	Mercury	Multiple	160/95	0.5 (13/2870)	0.4 (7/1653)	0.4 (20/4523)
Dash SC et al (1994) {B}	Orissa	>19	Yes	No	Random	Mercury	Multiple	160/95	NA	NA	2.6 (24/935)
Babu BV et al (1996) {A}	Andhra Pradesh	>19	NA	NA	NA	Mercury	Multiple	140/90	2.7 (4/148)	7.5 (11/147)	5.1 (15/295)
Babu BV et al (1996) {B}	Andhra Pradesh	>19	NA	NA	NA	Mercury	Multiple	140/90	1.4 (1/69)	5.9 (2/34)	2.9 (3/103)
Reddy BN et al (1998)	Andhra Pradesh	>17	No	No	Non-random	Digital	Multiple	160/95	0.0 (0/72)	0.0 (0/84)	0.0 (0/156)
Reddy KK et al (1999)	Kerala	>18	No	No	Non-random	Mercury	Multiple	140/90	NA	NA (NA/135)	2.6 (8/310)
Hazarika NC et al (2000) {A}	Assam	>29	No	No	Random	Mercury	Multiple	140/90	NA	NA	2.0 (2/98)
Hazarika NC et al (2000) {B}	Assam	>29	Yes	Yes	Random	Mercury	Multiple	140/90	NA	NA	11.8 (11/93)
Mukhopadhyay B et al (2001) {A}	Sikkim	>15	No	Yes	Non-random	Mercury	Multiple	160/95	31.9 (37/116)	25.0 (23/92)	28.8 (60/208)
Mukhopadhyay B et al (2001B)	Sikkim	>15	Yes	Yes	Non-random	Mercury	Multiple	160/95	31.9 (23/72)	27.3 (18/66)	29.7 (41/138)
Kusuma YS et al (2004) {A}	Andhra Pradesh	>20	No	No	Random	Mercury	Multiple	140/90	7.8 (9/115)	11.5 (13/113)	9.6 (22/228)
Kusuma YS et al (2004) {B}	Andhra Pradesh	>20	Yes	No	Random	Mercury	Multiple	140/90	13.5 (15/111)	32.4 (36/111)	23.0 (51/222)
NNMB Rural Report (2006) {A}	Kerala	>20	NA	NA	Random	Mercury	Multiple	140/90	80.0 (8/10)	40.0 (12/30)	50.0 (20/40)
NNMB Rural Report (2006) {B}	Tamil Nadu	>20	NA	NA	Random	Mercury	Multiple	140/90	10.0 (2/10)	8.7 (2/23)	9.3 (4/43)
NNMB Rural Report (2006) {C}	Karnataka	>20	NA	NA	Random	Mercury	Multiple	140/90	18.6 (24/129)	13.2 (25/189)	15.4 (49/318)
NNMB Rural Report (2006) {D}	Andhra Pradesh	>20	NA	NA	Random	Mercury	Multiple	140/90	23.5 (12/51)	19.1 (9/47)	21.4 (21/98)
NNMB Rural Report (2006) {E}	Maha-rashtra	>20	NA	NA	Random	Mercury	Multiple	140/90	30.8 (48/156)	11.2 (16/143)	21.4 (64/299)
NNMB Rural Report (2006) {F}	Gujarat	>20	NA	NA	Random	Mercury	Multiple	140/90	7.3 (15/206)	4.6 (12/262)	5.8 (27/468)
NNMB Rural Report (2006) {G}	Madhya Pradesh	>20	NA	NA	Random	Mercury	Multiple	140/90	16.5 (47/285)	17.0 (45/264)	16.8 (92/549)
NNMB Rural Report (2006) {H}	Orissa	>20	NA	NA	Random	Mercury	Multiple	140/90	40.9 (99/242)	44.8 (107/239)	42.8 (206/481)
NNMB Rural Report (2006) {I}	West Bengal	>20	NA	NA	Random	Mercury	Multiple	140/90	24.5 (24/98)	26.6 (25/94)	25.5 (49/192)
Ghosh R (2007)	West Bengal	>13	Yes	Yes	Random	Mercury	Single	140/90	NA	21.6† (22/102)	NA
Tiwari RR (2008)	Gujarat	>20	Yes	Yes	Random	Mercury	Multiple	140/90	16.5 (15/91)	17.5 (11/63)	16.9 (26/154)
Kusuma YS et al (2008)	Orissa	>17	NA	NA	Random	Mercury	Multiple	140/90	24.8 (32/129)	13.4 (18/134)	19.0 (50/263)
Kapoor AK et al (2008)	Uttaranchal	>20	No	No	Non-random	Mercury	Single	140/90	61.9‡ (39/63)	NA	NA
NNMB Tribal Report (2009) {A}	Madhya Pradesh	>19	NA	NA	Random	Mercury	Multiple	140/90	13.6 (12/88)	22.4 (30/134)	18.9 (42/222)
NNMB Tribal Report (2009) {B}	Madhya Pradesh	>19	NA	NA	Random	Mercury	Multiple	140/90	22.1 (270/1220)	25.5 (317/1234)	23.8 (587/2463)
NNMB Tribal Report (2009) {C}	Madhya Pradesh	>19	NA	NA	Random	Mercury	Multiple	140/90	29.1 (57/196)	26.1 (58/222)	27.5 (115/418)
NNMB Tribal Report (2009) {D}	Madhya Pradesh	>19	NA	NA	Random	Mercury	Multiple	140/90	7.4 (27/364)	10.5 (28/267)	8.7 (55/631)
NNMB Tribal Report (2009) {E}	Madhya Pradesh	>19	NA	NA	Random	Mercury	Multiple	140/90	32.8 (41/125)	33.8 (52/154)	33.3 (93/279)
NNMB Tribal Report (2009) {F}	Madhya Pradesh	>19	NA	NA	Random	Mercury	Multiple	140/90	8.1 (7/86)	12.8 (14/109)	10.8 (21/195)
NNMB Tribal Report (2009) {G}	Madhya Pradesh	>19	NA	NA	Random	Mercury	Multiple	140/90	25.4 (85/335)	26.5 (89/336)	25.9 (174/671)
NNMB Tribal Report (2009) {H}	Kerala	>19	NA	NA	Random	Mercury	Multiple	140/90	44.8 (847/1890)	35.8 (824/2302)	39.9 (1671/4192)
NNMB Tribal Report (2009) {I}	Tamil Nadu	>19	NA	NA	Random	Mercury	Multiple	140/90	17.8 (460/2586)	18.4 (721/3921)	18.2 (1182/6507)
NNMB Tribal Report (2009) {J}	Karnataka	>19	NA	NA	Random	Mercury	Multiple	140/90	28.4 (724/2551)	25.5 (1003/3935)	26.6 (1728/6486)
NNMB Tribal Report (2009) {K}	Andhra Pradesh	>19	NA	NA	Random	Mercury	Multiple	140/90	17.0 (577/3397)	20.8 (849/4083)	19.1 (1427/7480)
NNMB Tribal Report (2009) {L}	Maharashtra	>19	NA	NA	Random	Mercury	Multiple	140/90	27.7 (579/2089)	19.3 (436/2259)	23.3 (1015/4348)
NNMB Tribal Report (2009) {M}	Gujarat	>19	NA	NA	Random	Mercury	Multiple	140/90	9.9 (269/2717)	6.3 (218/3461)	7.9 (487/6178)
NNMB Tribal Report (2009) {N}	Orissa	>19	NA	NA	Random	Mercury	Multiple	140/90	53.7 (775/1443)	48.8 (691/1416)	51.3 (1466/2859)
NNMB Tribal Report (2009) {O}	West Bengal	>19	NA	NA	Random	Mercury	Multiple	140/90	29.9 (614/2053)	30.1 (728/2418)	30.0 (1342/4471)
Sachdev B et al (2011a)	Rajasthan	>17	NA	NA	NA	Digital	Multiple	140/90	NA	NA (NA/91)	19.7 (34/173)
Manimuda SP et al (2011)	Andaman & Nicobar Islands	>18	Yes	Yes	Random	Mercury	Multiple	140/90	50.7 (215/424)	50.3 (277/551)	50.5 (492/975)
Mungreiphy NK et al (2011)	Manipur	>19	Yes	No	Non-random	Mercury	Multiple	140/90	21.8‡ (56/257)	NA	NA
Sachdev B et al (2011b) {A}	Rajasthan	>17	NA	NA	NA	Digital	Single	140/90	NA	NA	27.1 (118/435)
Sachdev B et al (2011b) {B}	Rajasthan	>17	NA	NA	NA	Digital	Single	140/90	NA	NA	16.3 (68/418)
Sachdev B et al (2011b) {C}	Rajasthan	>17	NA	NA	NA	Digital	Single	140/90	NA	NA	22.7 (34/150)
Sachdev B et al (2011b) {D}	Rajasthan	>17	NA	NA	NA	Digital	Single	140/90	NA	NA	27.3 (15/55)
Sachdev B et al (2011b) {E}	Rajasthan	>17	NA	NA	NA	Digital	Single	140/90	NA	NA	19.4 (14/72)
Sachdev B et al (2011b) {F}	Rajasthan	>17	NA	NA	NA	Digital	Single	140/90	NA	NA	30.9 (34/110)
Sachdev B et al (2011b) {G}	Rajasthan	>17	NA	NA	NA	Digital	Single	140/90	NA	NA	21.7 (10/46)
Borah PK et al (2011)	Mizoram	>17	NA	NA	Random	Mercury	Multiple	140/90	34.4 (85/247)	20.7 (61/294)	27.0 (146/541)

Letters within ‘{}’ indicate subpopulations from the same study.

ST – Scheduled Tribe, NA – no information available.

* - Prevalence of hypertension in both sexes combined unless otherwise specified.

† - Study done exclusively in females.

‡ - Study done exclusively in males.

### Random effects pooled estimate

**Figure 2:**
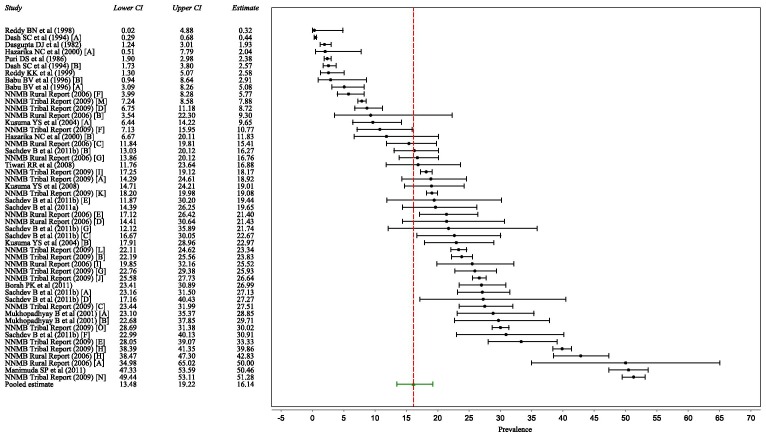
Forest plot of studies on hypertension prevalence.

The pooled estimate for mean prevalence of hypertension among adult tribal populations of India was 16.1%, 95% CI: 13.5% to 19.2% (table 2, figure 2). There was significant heterogeneity between the studies (I^2^ = 99% and Cochran's Q = 4624.0, df  = 49, p<0.001). The prevalence in females was lower than in males but the difference was not significant (18.6 vs 19.3%, p = 0.61, Figures S2 and S3).

**Table 2:** Random effects mean percent of hypertension by subgroup and sensitivity analyses.

		Mean percent	95% CI	I^2^	Cochran's Q	p
**All studies**		16.1	13.5, 19.2	98.8	4624.0	<0.001
**Subgroup analysis**						
Sex						
	Females	18.6	16.2, 21.2	98.1	0.3	0.618
	Males	19.3	15.7, 23.5			
Age (years)						
	≤45	11.0	4.8, 23.4	97.5	2.8	0.093
	>45	28.0	12.7, 50.9			
Time period						
	1981–1990	2.3	1.9, 2.8	98.8	305.1	<0.001
	1991–2000	2.4	1.0, 5.7			
	2001–2011	22.5	19.3, 26.2			
Region*						
	Himalayan & North-eastern	9.2	3.2, 24.1	98.8	1.7	0.411
	Southern	18.4	14.6, 22.9			
	Central	17.8	13.6, 22.9			
Status of acculturation						
	Not acculturated	2.8	0.9, 8.5	98.8	13.2	0.001
	Acculturated	17.7	6.7, 39.2			
	Unknown†	20.8	17.6, 24.5			
Special features						
	None	2.7	0.8, 8.3	98.8	13.5	0.001
	Yes‡	18.1	5.1, 47.7			
	Unknown†	20.8	17.6, 24.5			
BP apparatus						
	Mercury	15.3	12.5, 18.6	98.8	5.6	0.02
	Digital	22.0	17.6, 27.2			
Number of BP recordings						
	Multiple	15.1	12.4, 18.4	98.8	8.6	0.003
	Single	23.3	18.8, 28.4			
Cut-off used for classification (mm Hg)						
	160/95	3.5	0.94, 12.1	98.8	7.6	0.006
	140/90	19.6	16.6, 22.9			
Sampling strategy						
	Non-random scheme	6.1	1.3, 24.4	98.8	2.1	0.355
	Random scheme	17.1	14.0, 20.8			
	Unknown†	17.9	13.0, 24.1			
**Sensitivity analyses**						
	Removal of six low quality studies	16.4	13.3, 20.1	99.1	4466.1	<0.001
	Removal of five outlier subpopulations	14.1	12.0, 16.5	97.9	2349.9	<0.001
	Removal of five outlier subpopulations and study with zero prevalence	14.3	12.2, 16.7	97.9	2340.3	<0.001
	Removal of subpopulations with sample size less than 100	15.8	13.0, 19.1	99.0	4581.4	<0.001

All subgroup and sensitivity analyses were done only for both sexes combined except for the subgroup ‘sex’.

*Himalayan & north-eastern – Uttaranchal, Himachal Pradesh, Assam, Manipur, Mizoram & Sikkim, southern – Andhra Pradesh, Kerala, Karnataka, Tamil Nadu from mainland India & Andaman & Nicobar Islands, central – Gujarat, Rajasthan, Maharashtra, Madhya Pradesh, Orissa & West Bengal.

† Unknown – no information available.

‡ Special features include – consumption of large quantities of meat and milk products, prevalent use of alcohol containing drinks and/or tobacco, intake of large quantities of salt, salt tea or any other as stated by the authors.

### Subgroup analyses

**Figure 3:**
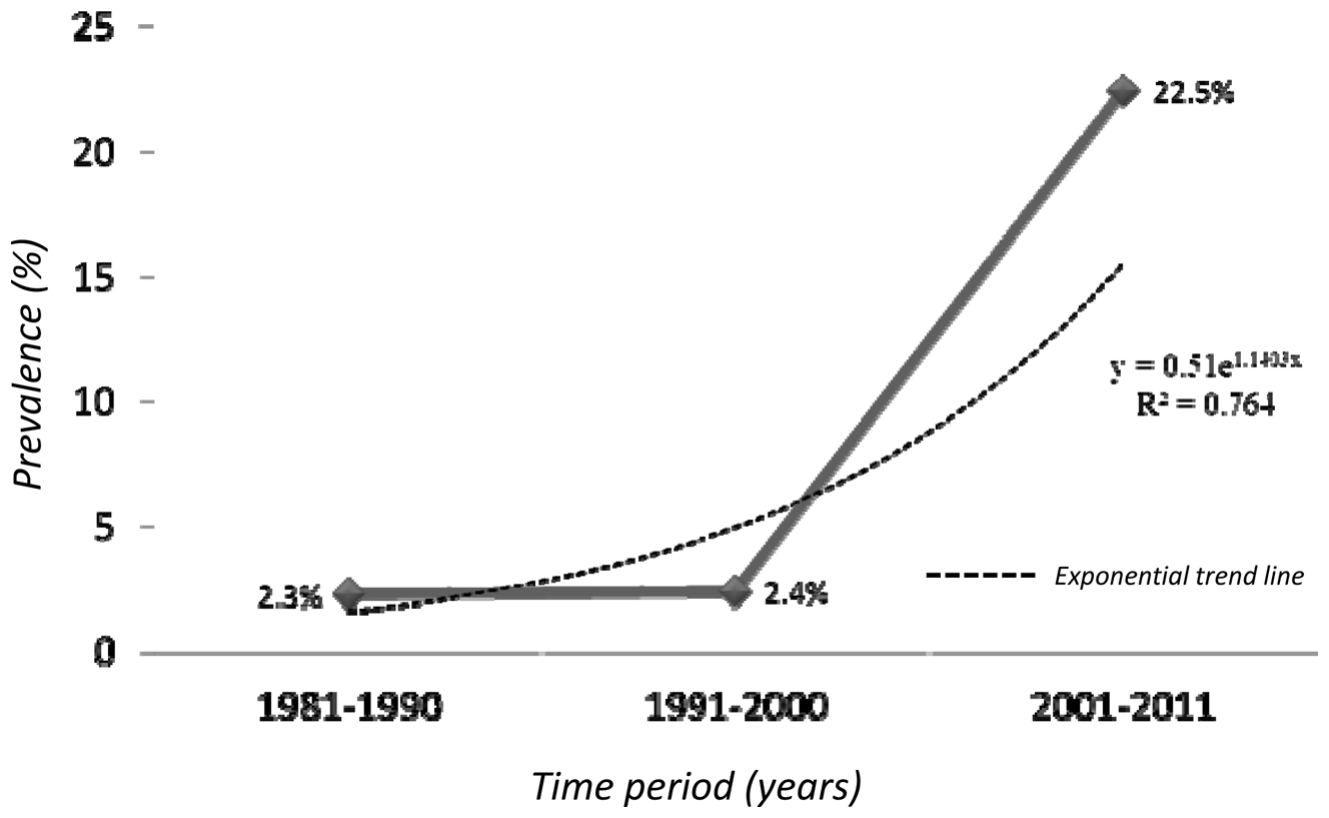
Trends in hypertension prevalence (1981–2011).

Studies done exclusively in males or females were excluded from subgroup and sensitivity analyses. Only five studies provided age wise prevalence, and their age categories were clubbed into two groups (≤45 and >45 years). In case of overlap, categorization was based on class interval midpoint. Age wise (Figure S4) and region wise differences were non-significant. Prevalence for the first (1981–1990) and second (1991–2000) decades were 2.3% (1.9 to 2.8) and 2.4% (1.0 to 5.7) respectively, which increased to 22.5% (19.3 to 26.2) in the third (2001–2011) decade. This increase was statistically significant (p<0.001, figure 3). A few studies provided information on lifestyle practices of tribes from which their acculturation status can be judged. The prevalence was significantly higher in tribes that were judged acculturated as compared to those that were not (17.7% vs. 2.8%, p = 0.001). Prevalence differed significantly between studies that used a mercury sphygmomanometer, and those which used a digital apparatus (15.3% vs 22.0%, p = 0.02), between studies which used single reading, and those which used multiple readings (23.3% vs. 15.1%, p = 0.003), and between studies that used the 160/95 mm Hg cut-off, and those which used the 140/90 mm Hg cut-off (3.5% vs. 19.6%, p = 0.006). Differences due to sampling schemes were non-significant (p = 0.34).

### Meta-regression analysis

Random effects meta-regression analysis done with covariates having a p value <0.20 in the subgroup analyses showed that ‘decade of study’ was the only significant covariate that independently and significantly affected the prevalence. The overall model was significant with an R^2^ = 0.57 and p value <0.001 (table 3).

**Table 3:** Random effects meta-regression analysis – effect of covariates on the prevalence of hypertension.

Covariate	Coefficient	95% CI	SE	Z	p value
Decade of study	1.630	1.10, 2.20	0.28	6.21	<0.001
Status of acculturation	0.004	−0.06, 0.08	0.03	0.14	0.81
BP apparatus	0.580	−0.74, 1.90	0.68	0.86	0.38
Number of BP recordings	−0.650	−2.07, 0.76	0.72	−0.90	0.36
Cut-off used for classification (mm Hg)	−0.160	−1.05, 0.72	0.45	−0.46	0.71
Constant	−6.140	−7.83, −4.45	0.86	−7.38	<0.001

Coefficient is for logit of proportion.

Dependent variable: prevalence of hypertension.

Reference categories of independent variables: decade 1981–1990, BP apparatus – digital, number of readings – single reading, cut-off used for hypertension −140/90 mm Hg, and status of acculturation – not acculturated.

### Sensitivity analyses

We reran the main analysis by removing one subpopulation at a time sequentially to identify specific sources of heterogeneity. The pooled estimates did not vary much from the original analysis during each removal (data not shown). Removal of six low quality studies or the smaller (size <100) subpopulations did not affect the original estimate. However, removing outlier subpopulations yielded a slightly lower estimate than the original (14.3% vs. 16.1%, table 2).

### Publication bias

Publication bias has a less important role in meta-analysis of prevalence studies. However, visual inspection of the funnel plot showed that some smaller studies displayed a large effect (which was accounted for by sensitivity analysis), and that there were more studies to the left of estimate than on the right (Figure S5). The Duval and Tweedie's trim and fill method, which looks for missing studies using random effects model and refills the plot, did not find evidence of publication bias. The adjusted values (16.6%, 95% CI: 13.9, 19.5) were no different from observed values (16.6%, 95% CI: 13.9, 19.5).

## Discussion

The adverse health effects of elevated BP have been recognized since the early part of 19^th^ century [21], [22], and population level studies in India had been conducted as early as the late 1940s [23], [24]. However tribal hypertension received very little attention prior to 2000. A relatively lower prevalence of hypertension among tribal populations has intrigued scientists around the world. Since tribal characteristics make them less amenable to research on a large scale, it was pertinent that this meta-analysis be undertaken. To the best of our knowledge this is the first systematic review and meta-analysis of hypertension prevalence among adult tribal populations of India.

Our estimated prevalence of hypertension in tribal populations was lower than the figures reported in general population for comparable time periods. There was no survey based country level estimate among general population for comparison with the current study's estimate. The Integrated Disease Surveillance Project study done among general population in seven states reported a prevalence ranging from 20% to 27% in urban areas and 18% to 26% in rural areas for 2007–08 [25]. Gupta R reported in a review that the prevalence reported in studies conducted after 1994 ranged from 14% to 45% in urban areas and 3% to 24% in rural areas [26]. According to WHO, the estimated prevalence for general population for 2008 was 32.5%, which was higher than our estimate of 22.5% among tribal populations for the period 2001–11 [27]. This showed that tribal populations were still at lower risk for having hypertension as compared to general population. Nonetheless, the increasing trend in tribal populations closely followed that in the general population, probably providing proof to the notion that even tribal populations were not immune to the health effects of modern lifestyles [28],[29]. However, the effect seemed to be delayed. The ‘early versus later adopter’ analogy that is often cited to explain differences between developed and developing nations in experiencing the NCD burden can be adapted to this scenario as well (tribal populations acting as late adopters) [30]. The apparent association between advancing time and increasing prevalence was probably driven by underlying modernisation. Tribes that were adjudged to be acculturated and to have special features (like prevalent consumption of increased salt and alcoholic beverages) displayed a higher prevalence. This finding was in line with the prevalent opinion that acculturation exposes traditional populations to diseases of modern lifestyle [31]–[35]. Studies conducted among tribes of South America, Malaysia, and Africa that had undergone acculturation have recorded prevalence in the range of 10 to 35% [36]–[39]. Although we couldn't find direct evidence against modernisation in this review, it can be postulated that modernisation has increased through the years and was responsible for the observed increase in trend. We stratified time into two periods (1981–2001 and 2001–2011) and estimated the prevalence in acculturated and unacculturated tribes, for each period separately. We found that the prevalence in unacculturated tribes increased from 1.8 to 22.7% and in acculturated tribes it increased from 4.5 to 27.5% between the two periods. The prevalence increased in both groups with almost equal intensity. This showed that other factors, which we were unable to address, were also probably responsible for the increase. This was supported by the regression model that was able to explain only half the variation in prevalence.

We tried to study several factors that affected hypertension. A few factors such as sex, age and region were non-significantly associated with hypertension. However, these important factors affect prevalence and need explanation. Although we found no sex difference, studies have examined the role of sex as a determinant of blood pressure and found lower prevalence among females [40], [41]. We found that hypertension was more prevalent, albeit non-significantly, in the older age group (>45 years) which was contrary to the conventional knowledge that blood pressure doesn't increase with age in tribal populations [4], [10], [32]. This finding was based on a small number of studies, and hence requires stronger evidence before we can draw conclusions. The lower prevalence in Himalayan and north-eastern region (higher altitude regions) as compared to other regions could be partly explained by altitudinal differences. The BP lowering effect of high altitudes is a well-established fact i.e., chronic hypoxic stress at high altitudes leads to vasodilation and consequent fall in BP [42], [43].

Some of the methodological factors were found to be significantly associated with prevalence in bivariate analysis. Unstandardized techniques of BP measurement have been known to cause misclassification bias, but international guidelines have led researchers to use uniform methodology lately [44]. As expected, studies that used a higher cut-off reported a lower prevalence. It should be noted here that studies that used higher cut-off values were also the ones which were done in earlier decades. However, this factor was adjusted in the meta-regression and decade of study was found to be only factor influencing the prevalence. Studies that used a random sampling scheme reported a slightly higher value than those that used a non-random scheme. Since characteristics of tribal populations (lack of sampling frame, difficult terrain and nomadic nature) inhibit random sampling, it is possible that health conscious individuals would more likely participate in a volunteer driven sampling, and thus explain the lowered estimate (i.e. healthy volunteer bias) in non-random samples.

### Strengths and limitations

The main advantage of this review was the large sample size which resulted in the highly precise pooled estimate. The review provided an opportunity to examine the influence of several candidate factors on the pooled estimate by means of meta-regression analysis. There was no evidence of publication bias, which further increased the confidence in our estimate. There were certain limitations as well. Firstly, many blood pressure studies in tribal populations could not be included due to non-reporting of prevalence (Table S4). Their inclusion might have shifted the estimate from the current value. Secondly, assessment of acculturation status was subjective. However, it was based on the consensus of at least two authors and misclassification would have been minimal. Thirdly, many studies did not provide information on acculturation status systematically, which probably reduced the reliability of this covariate. Fourthly, individual tribe level data for all studies could not be obtained. This forced us to use information at different levels of the population, which was probably one of the reasons for heterogeneity. Fifthly, estimates for earlier time periods were based on fewer studies when compared to estimates for later periods, which introduced variations in precision levels. But, since we were only concerned with the trend, this limitation can be safely ignored. Sixthly, studies that used complex sampling methods did not provide a weighted prevalence which could have caused errors in estimation. Seventhly, about 35 different tribes that were included in this review, constituted just 5% of all tribes present in the country. But considering the paucity of data this could be a useful starting point for future research. Eighthly, in meta-analysis of proportions there is a tendency for variances to be unstable and to account for this we corroborated the results of logit transformation with those of a different transformation technique (Freeman-Tukey) and found similar estimates. (Table S5) This exercise increases the credibility of estimates obtained here.

Finally, we tried to study many factors that affected hypertension prevalence, but several other factors and complex interactions between them were not explored. These must be given more focus in future research.

### Suggestions for future research

Future studies on tribal populations can be improved by addressing some of these issues. Apart from conventional factors, other factors like nomadic practices, acculturation status, and dietary pattern should be explored in detail in studies of tribal hypertension [34], [35]. Since tribes widely differ with respect to these characteristics, by ignoring them we may run the risk of over simplification by amalgamating the so called ‘tribal populations’ into a single group. An objective scale should be used to assess level of acculturation. Studies using non-random samples are seldom of use. There is a need for studies that are representative and with adequate sample size. Primitive tribes that are rarely investigated should be also be brought into focus. It is quite possible that tribes that are most affected remain the least studied.

### Implications

The evidently increasing trend in hypertension prevalence shall require concerned parties to initiate immediate curative and sustainable preventive measures to control this emerging health concern in tribal populations. Although acculturation seems to be the major responsible factor, there are other underlying factors that drive the increase. There is a need to obtain an in-depth understanding of these factors to better explain the rising trend. Among these, altitude, physical activity, social factors, economic status, diet, genetic factors, behavioural factors, body build, age structure of population, and access to health care are important.

## Conclusion

Apart from hypertension it has also been reported that prevalence of other NCDs like Diabetes Mellitus is also increasing among tribes [45]. These corroborating evidences highlight the vulnerability of tribal groups to illness states that were so far considered to affect only the well-off urban masses. It has to be recognised widely that tribes face newer emerging health problems, in addition to the conventional diseases [46]. The Government of India has committed itself to the advancement of such underserved groups through various schemes and had set up a dedicated ministry for tribal affairs. Findings of this study would help appraise concerned policy makers of the changing health needs of tribal communities in India.

### What is already known on this topic?

Studies documented lower prevalence of hypertension among tribal populations in India.

But some recent studies started reporting higher prevalence.

There has been no quantitative synthesis of prevalence of hypertension among tribal populations.

### What this study adds?

Tribes are experiencing higher burden of hypertension with advancing time.

This finding should lead policy makers to respond to emerging health issues of tribal populations.

Although this increase is partly explained by modernization, there are several unmeasured factors that need in-depth understanding.

## Supporting Information

Figure S1 **Map showing the three regions and exact locations of the studies (only 16 studies provided exact location details).**
(JPG)

Figure S2 **Forest plot of pooled estimate in males.**
(JPG)

Figure S3 **Forest plot of pooled estimate in females.**
(JPG)

Figure S4 **Forest plot of pooled estimates by age group.**
(JPG)

Figure S5 **Assessment of publication bias by funnel plot.**
(JPG)

Box S1 **PubMed search strategy.**
(DOCX)

Box S2 **Keywords used for searching other databases.**
(DOCX)

Box S3 **Quality assessment of studies included in the review.**
(DOCX)

Checklist S1 **Compliance with PRISMA/MOOSE guidelines.**
(DOCX)

Table S1 **Compliance with PRISMA/MOOSE guidelines.**
(DOCX)

Table S2 **Quality assessment of studies included in the review.**
(DOCX)

Table S3 **Characteristics of the tribal communities described in the studies.**
(DOCX)

Table S4 **Characteristics of studies that were excluded from review.**
(DOCX)

Table S5 **Pooled estimates derived by Freeman-Tukey transformation of proportions.**
(DOCX)

Text S1 **References of the articles included in the review.**
(DOCX)

Protocol S1 **Protocol for systematic review and meta analysis of hypertension in Indian tribes.**
(DOCX)
