# Correction: Prevalence of Hypertension in Indian Tribes: A Systematic Review and Meta-Analysis of Observational Studies

**DOI:** 10.1371/journal.pone.0109008

**Published:** 2014-09-17

**Authors:** The PLOS ONE Staff

The images for Figures S3 and S4 are incorrectly switched. The image that appears as Figure S3 should be Figure S4, and the image that appears as Figure S4 should be Figure S3. Please view the correct Figures S3 and S4 below.

There is an error in the Methodology. The penultimate sentence of the Literature search strategy section of the Methodology should read: Study selection criteria are shown in Box S4 and full search strategy is detailed in Boxes S1 and S2.

Box S4 is omitted from the list of Supporting Information. It can be viewed below.

## Supporting Information

Figure S3 **Forest plot of pooled estimate in females.**
(JPG)

Figure S4 **Forest plot of pooled estimates by age group.**
(JPG)

Box S4 **PubMed search strategy.**
(DOCX)

## References

[1] Rizwan SA, Kumar R, Singh AK, Kusuma YS, Yadav K, et al (2014) Prevalence of Hypertension in Indian Tribes: A Systematic Review and Meta-Analysis of Observational Studies. PLoS ONE 9(5): e95896 doi:10.1371/journal.pone.0095896 24797244 10.1371/journal.pone.0095896 PMC4010404

