# Supplementary figures and images for: Prevalence of Hypertension in Indian Tribes: A Systematic Review and Meta-Analysis of Observational Studies
Source: PLoS One. 2014 May 5;9(5):e95896. doi: 10.1371/journal.pone.0095896 (PMC4010404; doi:10.1371/journal.pone.0095896)

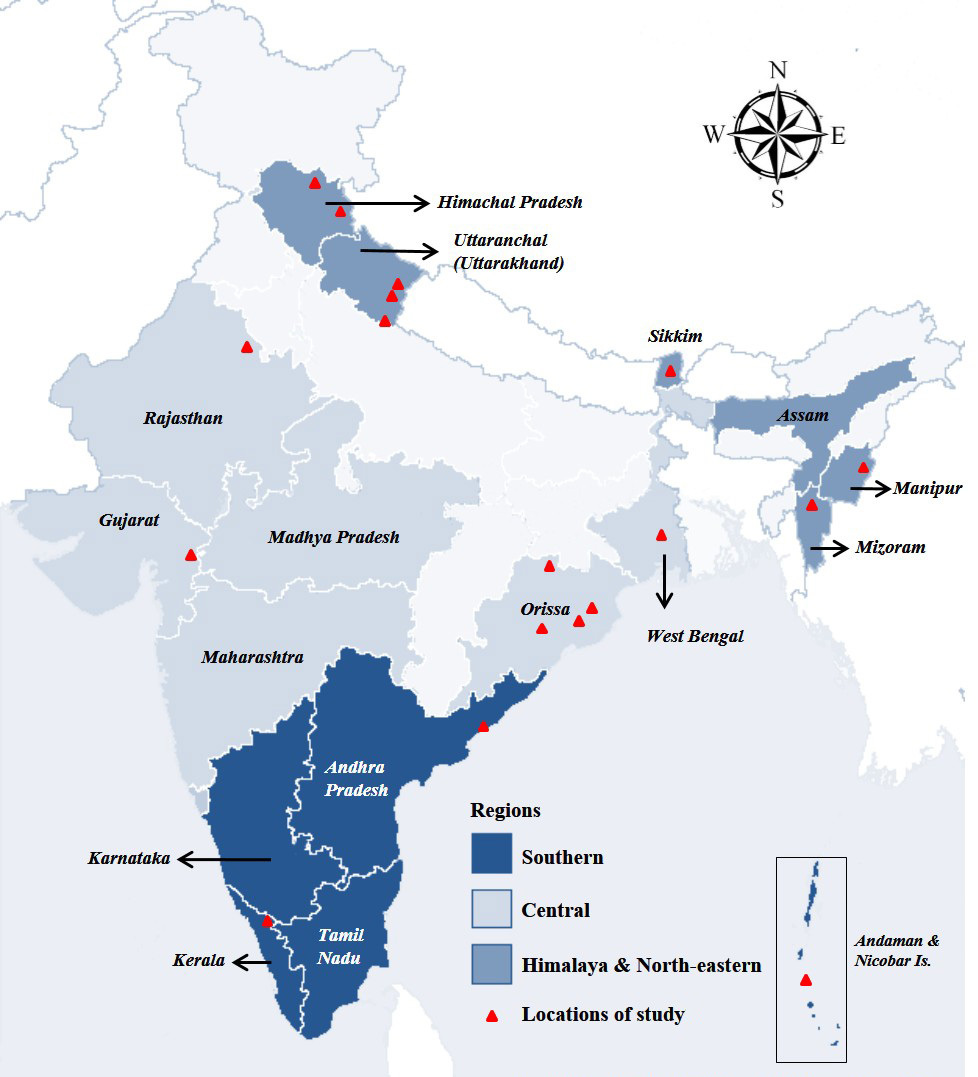

Supplement: Figure S1 — Map showing the three regions and exact locations of the studies (only 16 studies provided exact location details). (JPG) [file pone.0095896.s001.jpg]

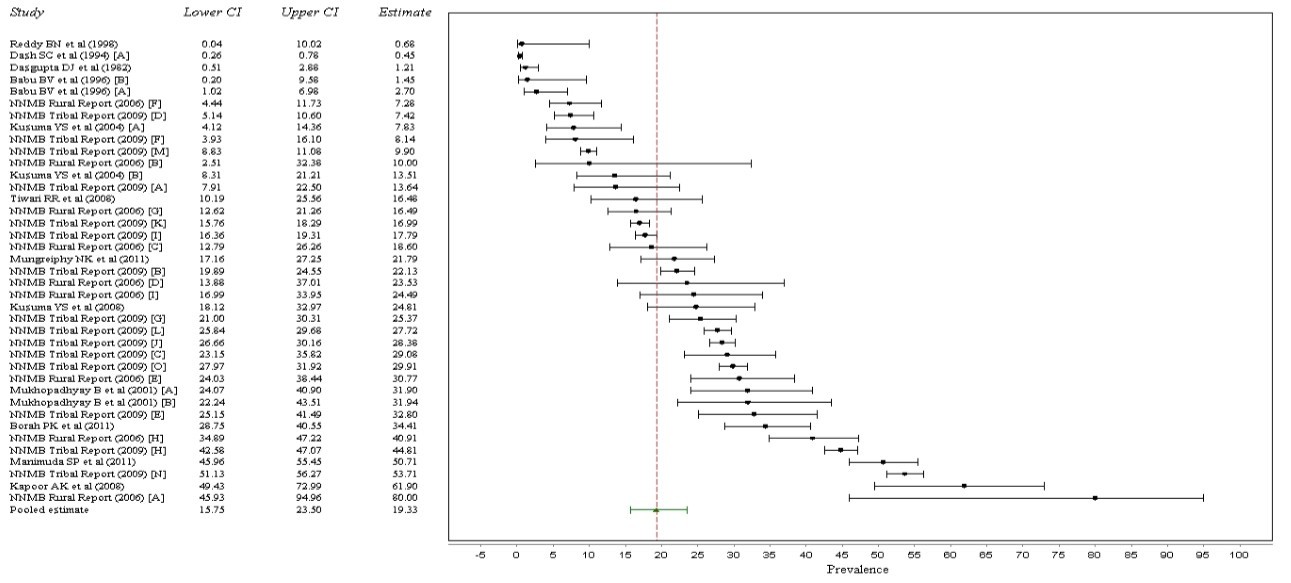

Supplement: Figure S2 — Forest plot of pooled estimate in males. (JPG) [file pone.0095896.s002.jpg]

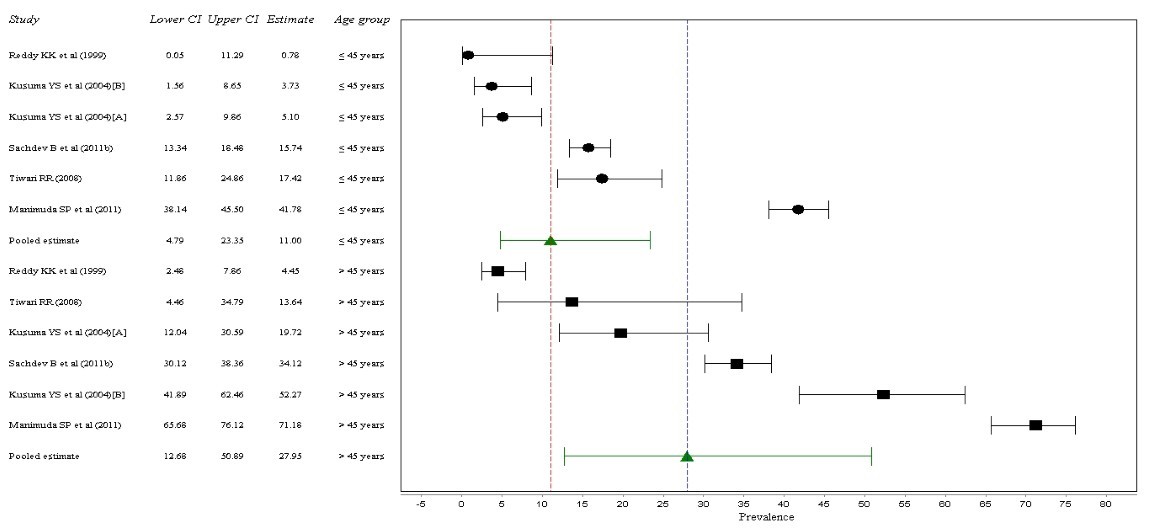

Supplement: Figure S3 — Forest plot of pooled estimate in females. (JPG) [file pone.0095896.s003.jpg]

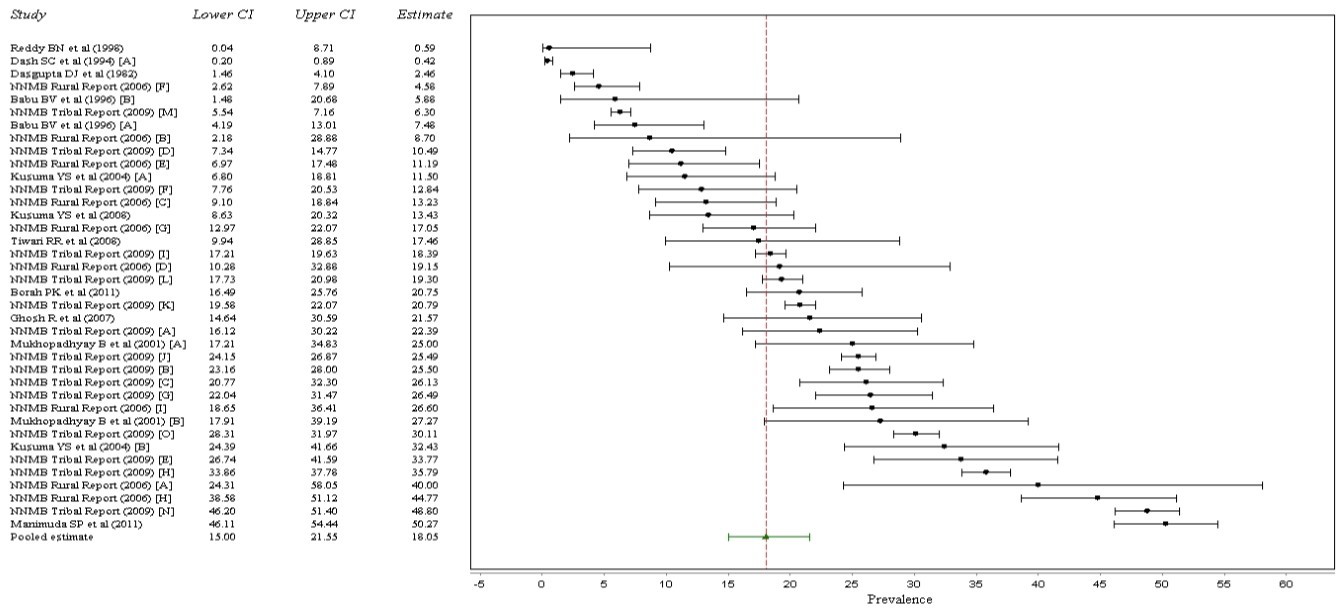

Supplement: Figure S4 — Forest plot of pooled estimates by age group. (JPG) [file pone.0095896.s004.jpg]

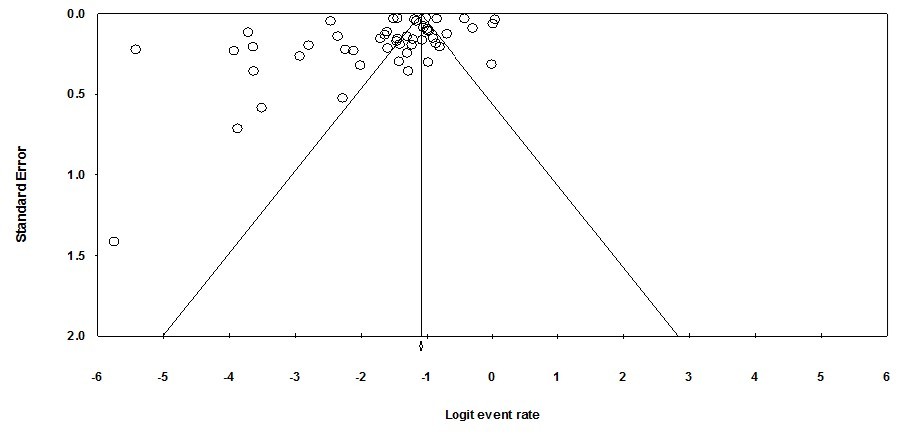

Supplement: Figure S5 — Assessment of publication bias by funnel plot. (JPG) [file pone.0095896.s005.jpg]
