# Supplementary material for: Prevalence of Hypertension in Indian Tribes: A Systematic Review and Meta-Analysis of Observational Studies
Source: PLoS One. 2014 May 5;9(5):e95896. doi: 10.1371/journal.pone.0095896 (PMC4010404; doi:10.1371/journal.pone.0095896)
Supplement: Box S1 — PubMed search strategy. (DOCX) [file pone.0095896.s006.docx]

**Box S1. PubMed search strategy**

| 1 | Hypertension   1. MESH term ‘Hypertension’ 2. {Essential hypertension or Primary hypertension or High blood pressure or Elevated blood pressure or Raised blood pressure or Blood pressure} 3. Combine 1 a) and 1 b) |
| --- | --- |
| 2 | Prevalence   1. MESH term ‘Prevalence’ 2. {Epidemiology or Risk} 3. Add 2 a) or 2 b) to 1 c) |
| 3 | Tribal populations   1. MESH term ‘Ethnic Groups’ 2. {Trib$ or tradition$ or adivasi or nomad$ or ethin$ or aborgin$ or primitive trib$ or autochthon$ or indigen$ or nativ$} 3. Add 3 a) or 3 b) to 2 c) |
| 4 | India   1. India$ 2. Add 4 a) to 3 c) |

$ = wild card
