# Supplementary material for: Prevalence of Hypertension in Indian Tribes: A Systematic Review and Meta-Analysis of Observational Studies
Source: PLoS One. 2014 May 5;9(5):e95896. doi: 10.1371/journal.pone.0095896 (PMC4010404; doi:10.1371/journal.pone.0095896)
Supplement: Box S2 — Keywords used for searching other databases. (DOCX) [file pone.0095896.s007.docx]

**Box S2. Keywords used for searching other databases**

| 1 | Hypertension, Essential hypertension, Primary hypertension, High blood pressure, Elevated blood pressure, Raised blood pressure, Blood pressure. |
| --- | --- |
| 2 | Prevalence, Epidemiology, Risk. |
| 3 | Tribe(s), Tribal(s), Traditional population(s), Adivasi, Nomad(s), Nomadic, Ethnic, Ethnicity, Aboriginal, Aborigine(s), Primitive tribal(s), Primitive tribe(s), Autochthonous, Autochthonic, Indigenous, Native(s). |
| 4 | India, Indian. |

These four groups of keywords were used in several combinations in IndMED, Web of Science and Google Scholar. In Google Scholar, titles from the first 50 pages were screened for relevance.
