# Supplementary material for: Prevalence of Hypertension in Indian Tribes: A Systematic Review and Meta-Analysis of Observational Studies
Source: PLoS One. 2014 May 5;9(5):e95896. doi: 10.1371/journal.pone.0095896 (PMC4010404; doi:10.1371/journal.pone.0095896)
Supplement: Box S3 — Quality assessment of studies included in the review. (DOCX) [file pone.0095896.s008.docx]

**Box S3. Quality assessment of studies included in the review**

| The major aim of quality assessment was to determine the potential for selection bias (eligibility criteria, sampling strategy, sample size, non-response rate and generalizability) and measurement bias (BP measurement techniques). These two biases are especially important in cross-sectional studies that aim to estimate prevalence. A total of 11 domains were assessed. A score of one was given for fulfilling conditions in each domain, 0.5 for partial fulfilment and zero otherwise. The maximum possible score was 11 and a study scoring seven or more was classified as high quality study and low quality study otherwise. | |
| --- | --- |
| Domain | Conditions |
| Objectives | Clearly stated objective |
| Tribe description | Description of the tribe(s), from which a judgement could be made about the acculturation level and special features that may affect hypertension prevalence. |
| Study settings | Description of the study setting |
| Eligibility criteria | Clear description of eligibility criteria for participants (such as age group, sex, place of residence, absence of any ailments). |
| Sampling strategy | Detailed description of the sampling scheme used, based on which the type of sampling (random or non-random) can be determined. Random sampling was given a score of one and zero otherwise. |
| Sample size adequacy | Description of sample size calculations. If this was not done the relative precision was calculated (assuming simple random sampling) from the study sample size and estimated proportion. If the relative precision was less than or equal to 20% of the point estimate a score of one was given and zero otherwise. |
| BP measurement techniques | Detailed description of BP measurement techniques (preparation of the subject before measuring BP, position of the subject, type of apparatus used, number of readings taken, which value was taken as final reading, classification cut-off used) or give a reference of the procedures followed. |
| Non-response rate | Mention of non-response rate. If it was less than 20%, a score of one was given and zero otherwise. |
| Descriptive analyses | Description of socio-demographic profile of participants (age, sex, occupation, literacy and income). |
| Outcome data | Number of subjects classified as having hypertension or atleast the proportion from which number can be back calculated. |
| Discussion of generalizability | Should provide insights as to whether the findings can be generalized to similar populations elsewhere (discussion on possible biases). |
