# Supplementary material for: Prevalence of Hypertension in Indian Tribes: A Systematic Review and Meta-Analysis of Observational Studies
Source: PLoS One. 2014 May 5;9(5):e95896. doi: 10.1371/journal.pone.0095896 (PMC4010404; doi:10.1371/journal.pone.0095896)
Supplement: Table S2 — Quality assessment of studies included in the review. (DOCX) [file pone.0095896.s011.docx]

**Table S2. Quality assessment of studies included in the review**

| **Study** | **Clear objectives** | **Tribe description** | **Study Setting** | **Eligibility criteria** | **Sampling strategy** | **Sample size adequacy** | **BP measurement techniques** | **Response rate** | **Descriptive analysis** | **Outcome data** | **Discussion of Generalizability** | **Quality** |
| --- | --- | --- | --- | --- | --- | --- | --- | --- | --- | --- | --- | --- |
| Dasgupta DJ et al (1982) | Yes | Yes | Yes | No | Random | No | Yes | Yes,>80% | Yes | Yes | No | High |
| Puri DS *et al* (1986) | Yes | Yes | Yes | Yes | Non-random | No | Yes | Yes,>80% | Yes | Yes | Yes | High |
| Dash SC *et al* (1994) | Yes | Yes | Yes | No | Random | No | Yes | No | Yes | Yes | Yes | High |
| Reddy BN *et al* (1998) | Yes | Yes | Yes | No | Non-random | No | Yes | No | No | Yes | In part | Low |
| Babu BV *et al* (1996) | Yes | In part | Yes | No | NA | No | No | No | No | Yes | No | Low |
| Reddy KK *et al* (1999) | Yes | Yes | No | Yes | Non-random | No | Yes | No | Yes | Yes | Yes | High |
| Hazarika NC *et al* (2000) | Yes | In part | In part | No | Random | No | Yes | No | Yes | Yes | Yes | High |
| Mukhopadhyay B *et al* (2001) | Yes | Yes | Yes | In part | Non-random | Yes | Yes | No | Yes | Yes | Yes | High |
| Kusuma YS *et al* (2004) | Yes | Yes | Yes | No | Random | Yes | Yes | No | Yes | Yes | Yes | High |
| NNMB Rural Report (2006) | Yes | No | Yes | Yes | Random | Yes | Yes | Yes,>80% | Yes | Yes | No | High |
| Ghosh R (2007) | Yes | Yes | Yes | In part | Random | No | Yes | No | No | Yes | Yes | High |
| Tiwari RR (2008) | Yes | In part | No | Yes | Random | No | Yes | No | Yes | Yes | Yes | High |
| Kapoor AK *et al* (2008) | Yes | Yes | Yes | No | Non-random | Yes | In part | Yes,>80% | Yes | Yes | Yes | High |
| Kusuma YS *et al* (2008) | Yes | No | Yes | No | Random | No | In part | Yes | Yes | Yes | No | Low |
| NNMB Tribal Report (2009) | Yes | No | Yes | Yes | Random | Yes | Yes | Yes,>80% | Yes | Yes | No | High |
| Manimunda SP *et al* (2011) | Yes | Yes | Yes | No | Random | Yes | Yes | Yes,<80% | Yes | Yes | Yes | High |
| Mungreiphy NK *et al* (2011) | No | Yes | Yes | No | Non-random | No | Yes | No | No | Yes | Yes | Low |
| Borah PK *et al* (2011) | Yes | No | Yes | No | Random | Yes | In part | No | Yes | Yes | No | Low |
| Sachdev B *et al* (2011a) | Yes | No | No | Yes | NA | No | In part | No | Yes | Yes | Yes | Low |
| Sachdev B *et al* (2011b) | Yes | No | No | No | NA | Yes | Yes | No | No | Yes | No | Low |

NA – no information available
