# Supplementary material for: Prevalence of Hypertension in Indian Tribes: A Systematic Review and Meta-Analysis of Observational Studies
Source: PLoS One. 2014 May 5;9(5):e95896. doi: 10.1371/journal.pone.0095896 (PMC4010404; doi:10.1371/journal.pone.0095896)
Supplement: Table S3 — Characteristics of the tribal communities described in the studies. (DOCX) [file pone.0095896.s012.docx]

**Table S3. Characteristics of the tribal communities described in the studies**

| Study | Tribe(s) description |
| --- | --- |
| Dasgupta *et al* (1982) & Puri DS *et al* (1986) | Mixed origin tribes (Vedic Aryans, Khash Aryans and Mongols):Have mongoloid features, social and cultural life influenced by Hinduism and Buddhism, rural in nature, occupation includes subsistence farming, staple diet is barley and buck wheat. Display hard manual labour, polyandrous society. |
| Dash SC *et al* (1994) | Oraon:Theyare a rural population with placid environment; diet is virtually free of fat and just adequate calorie content. |
| Babu BV *et al* (1996) | Manzai Mali and Bod Mali: Small agricultural groups |
| Reddy BN *et al* (1996) | Yerukala:A semi-nomadic tribe, occupation includes pig rearing, hunting, subsist largely on begging from villagers for food, have no formal education, men consume home distilled alcohol. |
| Reddy KK *et al* (1999) | Kurichias: They enjoy high longevity relatively free from age associated chronic problems; lifestyle pattern is changing drastically due to association with industrial population. Healthy food habits like ragi and meat as staple foods, regular intake of green leafy vegetables and tubers. They work daily in paddy fields and walk for hours in forest for hunting and rearing the cattle. Have collective ownership of land, less expensive life, limited ambitions and adequate economic security. |
| Hazarika NC *et al* (2000) | Mizo: They have ancestral origin in China, live in hilly regions; diet has low salt and oil, physically very active, hill climbers.  Assamese:Theylead a typical Indian lifestyle; have lower alcohol and salt consumption. |
| Mukhopadhyay B *et al* (2001) | Lepchas: Indigenous to Sikkim Himalaya, have mongoloid features, language ‘Rongring’ (Tibeto-Burman family), predominantly hunter-gatherers and shifting hill cultivators in the past, settled agriculture and non-agriculture occupation followed currently, profess Lamaist Buddhism, some have adopted Christianity, Monogamy is the marital norm. Dzongu Lepchas’ diet includes cereals, roots and tubers, vegetables, meat and fish, prevalent consumption of indigenous millet beer (Chi) and high salt intake, use animal fat as cooking medium. Gangtok Lepchas have adopted urban way of life with better education, prevalent consumption of indigenous millet beer (Chi) and high salt intake. |
| Kusuma YS *et al* (2004) | Khondh: A primitive tribe, located in densely wooded hilltops, practice shifting cultivation and traditional agriculture practices, very low literacy, dialect ‘Kui’, dress in traditional way, have less access to modern transport, education, communication and medical facility.  Valmiki: Have lowest status in tribal social hierarchy, good access to modern amenities, highest literacy among tribes of Andhra Pradesh, dialect ‘Adivasi Oriya’, acculturated; occupation includes agriculture, forest labourer, petty trade, shifting cultivation with modern techniques, have economically better position. |
| Ghosh R (2007) | Munda: An Austro-Asiatic tribe, mostly live in remote areas like forests and mountains, depend on forest products and cultivation for livelihood, women engage in agricultural labour as daily wagers, poverty is prevalent, Munda women drink ‘Handia’ (prepared from fermented rice) with alcohol from a very young age, spend a large amount of income on alcohol. |
| Kapoor AK *et al* (2008) | Raji: A very underdeveloped Primitive Tribal Group, have very low literacy, are in a transitional stage between hunter-gatherer and pre-agricultural economy, some are labourers, display strenuous habitual physical activity, staple food is rice with wild vegetables like tubers, habitat located deep inside forests and approachable only by foot, social life is characterised by individualistic pattern, still follow nomadic life, have primitive mind-set and cultural belief, follow nuclear family and Hindu by religion. |
| Tiwari RR (2008) | Labourer tribal population |
| Manimunda SP *et al* (2011) | Nicobarese aborigines: Have high literacy, live in extended joint families, or ‘tuhets’, occupation includes cultivation of coconut, areca nut, banana and pig rearing, prevalent use of smokeless tobacco and alcohol (toddy from palm trees), undergoing acculturation and nutrition transition. |
| Mungreiphy NK *et al* (2011) | Tangkhul Naga: One of the subgroups of Naga tribe, belong to Mongoloid stock and Tibeto-Burman language family, influenced by Christianity and western education, staple food is rice, consume less sweet and oily food but lot of non-vegetarian food and leafy vegetables, growing fast food culture among young people; the tribe has undergone changes in occupation, economy, religion and overall lifestyle. |

Other studies included in the review did not provide description about the tribes studied.
