# Supplementary material for: Prevalence of Hypertension in Indian Tribes: A Systematic Review and Meta-Analysis of Observational Studies
Source: PLoS One. 2014 May 5;9(5):e95896. doi: 10.1371/journal.pone.0095896 (PMC4010404; doi:10.1371/journal.pone.0095896)
Supplement: Table S4 — Characteristics of studies that were excluded from review. (DOCX) [file pone.0095896.s013.docx]

**Table S4. Characteristics of studies that were excluded from review**

| **Author**  **(year of publication)** | **State** | **Age group** | **Tribe** | **Status of acculturation** | **Special features** | **Sampling scheme** | **BP apparatus** | **No of readings** | **Cut-off** | **Total no of males** | **No of male Hypertensives** | **Total no of females** | **No of female Hypertensives** | **Total sample** | **Total no of Hypertensives** | **Reason for exclusion** |
| --- | --- | --- | --- | --- | --- | --- | --- | --- | --- | --- | --- | --- | --- | --- | --- | --- |
| Solanki DM (Thesis) (1986) | Gujarat | NA | NA | NA | NA | NA | NA | NA | NA | NA | NA | NA | NA | NA | NA | Inadequate information to calculate prevalence |
| Dash SC *et al* (1986) | Orissa | 20-80 | Oraon | No | No | Random | Mercury | Multiple | 160/95 | 2870 | 13 | 1653 | 7 | 4523 | 20 | Repetition of Dash SC *et al* (1994) |
| Verma S *et al* (1987) | West Bengal | NA | Lodha | NA | NA | NA | NA | NA | NA | NA | NA | NA | NA | NA | NA | Inadequate information to calculate prevalence |
| Pandey AK *et al* (1991) | Jammu and Kashmir | 11-18 | Bod girls | No | No | Non-random | Mercury | Multiple | NA | NA | NA | 317 | NA | NA | NA | Age group not appropriate |
| Putatunda S *et al* (1994) | West Bengal | 20-65 | NA | NA | No | Random | Mercury | Multiple | NA | NA | NA | 75 | NA | NA | NA | Inadequate information to calculate prevalence |
| Mukhopadhyay B *et al* (1996) | Sikkim | >19 | Lepchas | No | Yes | Non-random | Mercury | Multiple | 160/95 | 117 | 36 | 97 | 25 | 214 | NA | Repetition of Mukhopadhyay B *et al* (2001) |
| Kusuma YS *et al* (2001) | Andhra Pradesh | >20 | Khondh,Valmiki | Mixed | No | Random | Mercury | Multiple | 140/90 | 646 | 125 | 670 | 144 | 1316 | 269 | Repetition of Kusuma YS *et al* (2004) |
| Kusuma YS *et al* (2002) | Andhra Pradesh | >20 | Khondh,Valmiki | Mixed | No | Random | Mercury | Multiple | 140/90 | 646 | 125 | 670 | 144 | 1316 | 269 | Repetition of Kusuma YS *et al* (2004) |
| Patni S *et al* (2003) | Uttaranchal | 6-68 | Bhoksa | No | No | Non-random | Mercury | NA | NA | 151 | NA | 135 | NA | 286 | NA | Inadequate information to calculate prevalence |
| Sarkar S *et al* (2006) {A} | West Bengal | >20 | Toto (R) | No | Yes | Random | Mercury | Multiple | 130/85 | 127 | NA | 131 | NA | 258 | NA | Inadequate information to calculate prevalence |
| Sarkar S *et al* (2006) {B} | West Bengal | >20 | Bhutia (R) | Yes | Yes | NA | Mercury | Multiple | 130/85 | 102 | NA | 128 | NA | 230 | NA | Inadequate information to calculate prevalence |
| Sarkar S *et al* (2006) {C} | West Bengal | >20 | Bhutia (U) | Yes | Yes | NA | Mercury | Multiple | 130/85 | 29 | NA | 46 | NA | 75 | NA | Inadequate information to calculate prevalence |
| Sarkar S *et al* (2008) | Sikkim | >20 | Bhutia (U) | Yes | Yes | Random | Mercury | Multiple | 140/90 | 100 | NA | 100 | NA | 200 | NA | Inadequate information to calculate prevalence |
| Kerketta AS *et al* (2009) {A} | Orissa | >60 | Dongria Kondh | No | No | Random | Mercury | Multiple | 140/90 | 30 | 4 | 20 | 5 | 50 | 9 | Age group not appropriate |
| Kerketta AS *et al* (2009) {B} | Orissa | >60 | Kutia Kondh | No | No | Random | Mercury | Multiple | 140/90 | 46 | 9 | 66 | 17 | 112 | 26 | Age group not appropriate |
| Kerketta AS *et al* (2009) {C} | Orissa | >60 | Langia Saora | Yes | No | Random | Mercury | Multiple | 140/90 | 20 | 13 | 30 | 20 | 50 | 33 | Age group not appropriate |
| Kerketta AS *et al* (2009) {D} | Orissa | >60 | Paudi Bhuiyan | No | No | Random | Mercury | Multiple | 140/90 | 39 | 8 | 61 | 24 | 100 | 32 | Age group not appropriate |
| Kapoor S *et al* (2010) | Madhya Pradesh | >17 | Saharia | No | No | Non-random | Mercury | NA | NA | 168 | NA | 196 | NA | 364 | NA | Inadequate information to calculate prevalence |
| Mishra SK *et al* (2010) | Sikkim | NA | Bhutia | NA | NA | NA | NA | NA | NA | NA | NA | NA | NA | 315 | NA | Inadequate information to calculate prevalence |
| Madani B *et al (*2011) | Gujarat | >20 | Naika, Rathwa, Damor | Yes | Yes | Random | Mercury | Multiple | 140/90 | 91 | 15 | 63 | 11 | 154 | 26 | Repetition of Tiwari RR *et al* (2008) |

Letters within ‘{}’ indicate subpopulations from the same study.

NA – no information available, R – rural, U – urban.
