# Supplementary material for: Prevalence of Hypertension in Indian Tribes: A Systematic Review and Meta-Analysis of Observational Studies
Source: PLoS One. 2014 May 5;9(5):e95896. doi: 10.1371/journal.pone.0095896 (PMC4010404; doi:10.1371/journal.pone.0095896)
Supplement: Table S5 — Pooled estimates derived by Freeman-Tukey transformation of proportions. (DOCX) [file pone.0095896.s014.docx]

**Table S5. Pooled estimates derived by Freeman-Tukey transformation of proportions**

|  | | **Mean percent** | **95% CI** |
| --- | --- | --- | --- |
| **All studies** | | 17.8 | 13.8, 22.2 |
| **Subgroup analyses** | | | |
| Sex | |  |  |
|  | Females | 19.0 | 14.8, 23.6 |
|  | Males | 20.9 | 15.7, 26.5 |
| Time period | | | |
|  | 1981-1990 | 2.2 | 1.8, 2.7 |
|  | 1991-2000 | 2.7 | 1.0, 5.0 |
|  | 2001-2011 | 23.3 | 19.7, 27.2 |
| Region* | | | |
|  | Himalayan & North-eastern | 12.2 | 4.2, 23.5 |
|  | Southern | 16.8 | 11.9, 22.4 |
|  | Central | 19.9 | 13.7, 27.0 |
| Status of acculturation | | | |
|  | Not acculturated | 3.9 | 1.5, 7.3 |
|  | Acculturated | 20.4 | 4.1, 44.8 |
|  | Unknown^†^ | 20.8 | 17.6, 24.5 |
| Special features | | | |
|  | None | 3.6 | 1.2, 7.3 |
|  | Yes^‡^ | 21.2 | 3.3, 48.8 |
|  | Unknown^†^ | 21.6 | 17.9, 25.5 |
| BP apparatus | | | |
|  | Mercury | 17.6 | 13.2, 22.5 |
|  | Digital | 18.8 | 11.3, 27.8 |
| Number of BP recordings | | | |
|  | Multiple | 17.0 | 12.7, 21.7 |
|  | Single | 23.5 | 18.8, 28.4 |
| Cut-off used for classification (mm Hg) | | | |
|  | 160/95 | 5.5 | 2.2, 10.1 |
|  | 140/90 | 20.4 | 16.9, 24.0 |
| Sampling scheme | | | |
|  | Non-random scheme | 8.7 | 1.4, 21.2 |
|  | Random scheme | 19.2 | 14.5, 24.5 |
|  | Unknown^†^ | 18.2 | 12.1, 25.3 |

The point estimates and confidence intervals are similar to the results obtained from employing a logit transformation.

Reference for the method of transformation: <http://www.statsdirect.com/help/default.htm#meta_analysis/proportion.htm>
