# Supplementary material for: Prevalence of Hypertension in Indian Tribes: A Systematic Review and Meta-Analysis of Observational Studies
Source: PLoS One. 2014 May 5;9(5):e95896. doi: 10.1371/journal.pone.0095896 (PMC4010404; doi:10.1371/journal.pone.0095896)
Supplement: Text S1 — References of the articles included in the review. (DOCX) [file pone.0095896.s015.docx]

T**ext S1: References of the articles included in the review**

1. Kusuma YS, Babu BV, Naidu JM. Prevalence of hypertension in some cross-cultural populations of Visakhapatnam district, south India. Ethn Dis. 2004;14:250–9.
2. Manimunda SP, Sugunan AP, Benegal V, Balakrishna N, Rao MV, Pesala KS. Association of hypertension with risk factors & hypertension related behaviour among the aboriginal Nicobarese tribe living in Car Nicobar Island, India. Indian J Med Res. 2011;133:287–93.
3. Mukhopadhyay B, Mukhopadhyay S. Blood pressure and its biocultural correlates among the Lepchas of Sikkim, India: a microlevel epidemiological study. Coll Antropol. 2001;25:97–110.
4. Borah P, Kalita H, Hazarika D, Shankarishan P, Mahanta J. Distribution of Plasma Homocysteine Concentration and Risk of Hypertension in a Tribal Population from North East India. Assam Journal of Internal Medicine. 2011;1:7–14.
5. Babu B, Kusuma Y, Naidu J. Distribution of blood pressure and influence of subcutaneous fat on systolic and diastolic levels in a tribal population. J Indian Med Assoc. 1996;94:289–93.
6. Dasgupta D, Prasher B, Vaidya N, Ahluwalia S, Sharma P, Puri D, Mehrotra AN. Blood pressure in a community at high altitude (3000m) at Pooh (North India). J Epidemiol Community Health. 1982;36:251–5.
7. Dash S, Sundaram K, Swain P. Blood pressure profile, urinary sodium and body weight in the “Oraon” rural and urban tribal community. J Assoc Physicians India. 1994;42:878–80.
8. Ghosh R. Microlevel determinants of blood pressure among women of two ethnic groups in a periurban area of Kolkata city, India. Am J Hum Biol. 2007;19:409–15.
9. Hazarika NC, Biswas D, Narain K, Phukan RK, Kalita HC, Mahanta J. Differences in Blood Pressure Level and Hypertension in Three Ethnic Groups of Northeastern India. Asia Pac J Public Health. 2000;12:71–8.
10. Kapoor AK, Tyagi R, Kapoor S. Nutritional status and cardio-respiratory functions among adult Raji males, a hunter and gatherer tribe of the Indian Himalayas. Anthropological Science. 2009;117:1–7.
11. Kusuma YS, Das PK. Hypertension in Orissa, India: a cross-sectional study among some tribal, rural and urban populations. Public Health. 2008;122:1120–3.
12. Mungreiphy NK, Kapoor S, Sinha R. Association between BMI, Blood Pressure, and Age: Study among Tangkhul Naga Tribal Males of Northeast India. Journal of Anthropology. 2011;2011:1–6.
13. National Nutrition Monitoring Bureau, National Institute of Nutrition Indian Council of Medical Research. Diet and Nutritional Status of Population and Prevalence of Hypertension among Adults in Rural Areas. Hyderabad, India;2006.
14. National Nutrition Monitoring Bureau, National Institute of Nutrition Indian Council of Medical Research. Diet and Nutritional Status of Tribal Population and Prevalence of Hypertension among Adults - Report on Second Repeat Survey. Hyderabad, India;2009.
15. Puri DS, Pal LS, Gupta BP, Swami HM, Dasgupta DJ. Distribution of blood pressure and hypertension in healthy subjects residing at high altitude in the Himalayas. J Assoc Physicians India. 1986;34:477–9.
16. Reddy BN. Blood pressure and adiposity: a comparative study of socioeconomically diverse groups of Andhra Pradesh, India. Am J Hum Biol. 1998;10:5–21.
17. Reddy KK, Rao AP, Reddy TP. Serum vitamins E, A and lipid peroxidation levels in Kurichias, an Indian tribal population. Indian J Biochem Biophys. 1999;36:44–50.
18. Sachdev B. Prevalence of hypertension and associated risk factors among nomad tribe groups. Antrocom Online Journal of Anthropology. 2011;7:181–9.
19. Sachdev B. Community based study on incidence of type 2 diabetes and hypertension among nomad tribal population of Rajasthan, India. International Journal of Science and Nature. 2011;2:296–301.
20. Tiwari RR. Hypertension and epidemiological factors among tribal labour population in Gujarat. Indian J Public Health. 2008;52:144–6.
