# Supplementary material for: Prevalence of Hypertension in Indian Tribes: A Systematic Review and Meta-Analysis of Observational Studies
Source: PLoS One. 2014 May 5;9(5):e95896. doi: 10.1371/journal.pone.0095896 (PMC4010404; doi:10.1371/journal.pone.0095896)
Supplement: Protocol S1 — Protocol for systematic review and meta analysis of hypertension in Indian tribes. (DOCX) [file pone.0095896.s016.docx]

**Protocol for Sytematic review and meta-analysis**

| Review title and timescale | | |
| --- | --- | --- |
| 1 | Review title Hypertension: new lessons from a systematic review and meta-analysis of prevalence studies in Indian tribes | |
| 2 | Original language titleEnglish. | |
| 3 | Anticipated or actual start date 02/04/2012 | |
| 4 | Anticipated completion date 31/05/2013 | |
| Review team details | | |
| 5 | | Named contactThe named contact acts as the guarantor for the accuracy of the information presented. Rizwan S A |
| 6 | | Named contact email sarizwan1986@gmail.com |
| 7 | | Named contact address PG Room, Ground floor, Centre for Community Medicine, Old OT block, All India Institute of Medical Sciences, New Delhi, Delhi - 110029, India. |
| 8 | | Named contact phone number +91 8447284098 |
| 9 | | Organisational affiliation of the review All India Institute of Medical Sciences, New Delhi Website address: http://www.aiims.edu/ |
| 10 | | Review team members and their organisational affiliations |
| 11 | | \| Title \| First name \| Last name \| Affiliation \| \| --- \| --- \| --- \| --- \| \| Dr \| Rizwan \| S A \| All India Institute of Medical Sciences, New Delhi \| \| Dr \| Arvind Kumar \| Singh \| All India Institute of Medical Sciences, New Delhi \| \| Dr  Dr \| Rakesh  Kusuma \| Kumar  Y S \| All India Institute of Medical Sciences, New Delhi  All India Institute of Medical Sciences, New Delhi \| \| Dr \| Kapil \| Yadav \| ICCIDD \| \| Professor \| Chandrakant S \| Pandav \| All India Institute of Medical Sciences, New Delhi \| |
| 12 | | Funding sources/sponsors None |
| 13 | | Conflicts of interest None known |
| 14 | | Collaborators |
|  | | None. |
| Review methods | | |
| 15 | | Review question(s) What is the estimated prevalence of hypertension (essential) among tribal populations of India? |
| 16 | | Searches MEDLINE (via PubMed), IndMED, Web of Science and Google Scholar. Articles published between 1st January 1981 and 31st December 2011. |
| 18 | | Condition or domain being studied Hypertension |
| 19 | | Participants/population Inclusion criteria: The study population being tribe(s) (tribe as declared by respective study authors or as mentioned in Scheduled Tribe list of the Article 342 of the Constitution of India for the given state). Study population should be apparently healthy, geographically and temporally well defined. Exclusion criteria: Study population exclusively includes age groups less than 12 years or more than 60 years. |
| 20 | | Intervention(s), exposure(s) None |
| 21 | | Comparator(s)/control None |
| 22 | | Types of study to be included initially Primary research, cross-sectional study or data, or first phase of a longitudinal study. |
| 24 | | Primary outcome(s) Prevalence of hypertension |
| 25 | | Secondary outcomes Factors affecting the prevalence of hypertension |
| 26 | | Data extraction, (selection and coding) All relevant data and study characteristics will be extracted onto a pre-coded spread sheet in Microsoft Office Excel independently by the two authors (RSA, RK). Information extracted will be first author, year of publication (taken to represent year of study), BP apparatus used, number of BP readings taken, cut-off used for hypertension classification, age group, place of study (state), name, status of acculturation & special features of the tribe, sampling scheme, sample size and number of hypertensives identified (separately for males and females). Data will be extracted at the lowest possible disaggregate level. If tribe wise disaggregate data are not available, the next highest level will be taken to represent a tribe. In case numbers of hypertensives are not mentioned, it will be calculated from the proportion. Disagreements in the data extracted will be resolved by discussion or by opinion of a third author (AKS). |
| 27 | | Risk of bias (quality) assessmentUsing appropriately modified critical appraisal checklists (like STROBE) each article will assessed for quality by two authors independently and disagreements in quality assessment will be resolved by third author. Sub group analysis by quality of studies will be done to remove the influence of low quality studies. |
| 28 | | Strategy for data synthesisPrimary analyses The effect size of interest for this meta-analysis will be the proportion of subjects classified as having hypertension. Analyses will be done using Comprehensive Meta-Analysis version 2.2 (Biostat, Englewood, NJ), Stata/IC version 11.1 (StataCorp LP, College Station, TX) and Forest Plot Viewer software, version 1.0 (SRA International, Inc.). Heterogeneity between studies will be examined using I2 statistics and Cochran’s Q test for heterogeneity. All pooled estimates will be calculated using DerSimonian-Laird random effects model and reported as a proportion with 95% confidence intervals. Publication bias will be assessed by visual inspection of funnel plots and Duval and Tweedie's trim and fill technique. (42) Statistical significance is set at p value < 0.05. A meta-regression will be performed to determine the effect of covariates on the prevalence of hypertension by using meta-analytic random intercept, fixed slopes regression analysis (maximum likelihood estimation method). The regressions coefficients and their 95% confidence intervals will be presented. Sensitivity analyses We plan to perform sensitivity analyses by removing studies that are assessed to be low quality, by removing outlier studies whose point estimates are beyond three standard deviations and by removing studies that have sample size of less than 100, employing one strategy at a time. Time trend For estimating the secular trend, the entire period of 31 years i.e. from 1981 to 2011 will be divided into three decades i.e. 1981–1990, 1991–2000 and 2001-2011. Point estimates will then be calculated for each decade and plotted in a graph. |
| 29 | | Analysis of subgroups or subsetsSubgroup analyses Subgroup analyses will be done by sex, age, time period, region, status of acculturation, special features, procedures of BP recording, cut-offs used for hypertension classification and sampling strategy. |
| Review general information | | |
| 30 | | Type of review Epidemiologic |
| 32 | | Country India |
| 33 | | KeywordsGive words or phrases that best describe the review. (One word per box, create a new box for each term) Hypertension  Prevalence  Tribal  India |
