# Supplementary figures and images for: Correction: Prevalence of Hypertension in Indian Tribes: A Systematic Review and Meta-Analysis of Observational Studies
Source: PLoS One. 2014 Sep 17;9(9):e109008. doi: 10.1371/journal.pone.0109008 (PMC4168279; doi:10.1371/journal.pone.0109008)

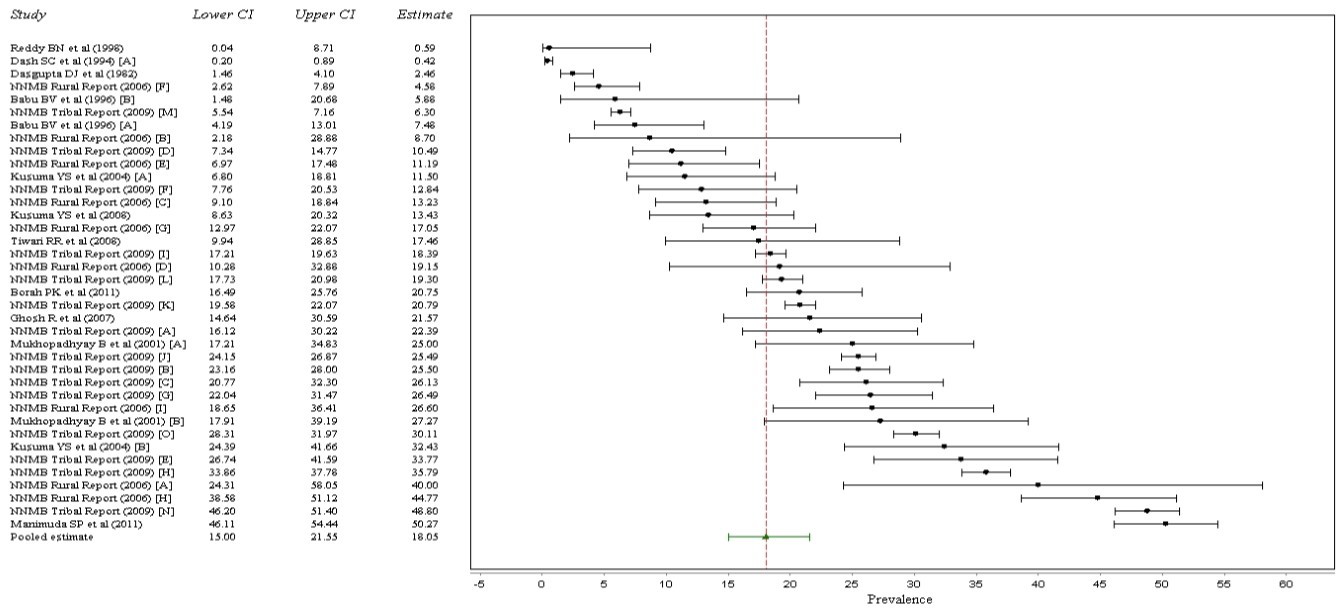

Supplement: Figure S3 — Forest plot of pooled estimate in females. (JPG) [file pone.0109008.s001.jpg]

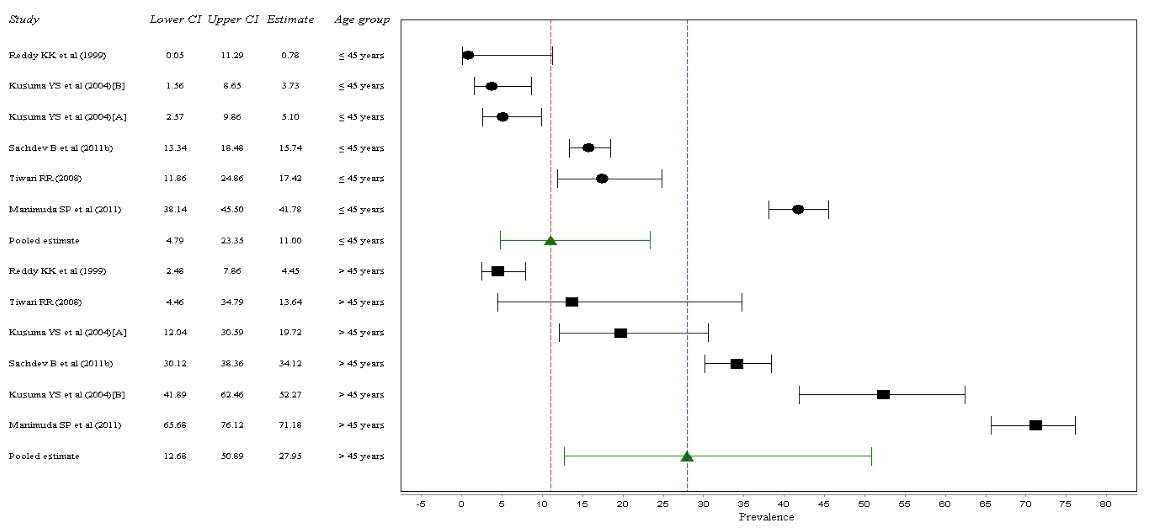

Supplement: Figure S4 — Forest plot of pooled estimates by age group. (JPG) [file pone.0109008.s002.jpg]
