# Supplementary material for: Correction: Prevalence of Hypertension in Indian Tribes: A Systematic Review and Meta-Analysis of Observational Studies
Source: PLoS One. 2014 Sep 17;9(9):e109008. doi: 10.1371/journal.pone.0109008 (PMC4168279; doi:10.1371/journal.pone.0109008)
Supplement: Box S4 — PubMed search strategy. (DOCX) [file pone.0109008.s003.docx]

Box S4 Study selection criteria

| 1. Inclusion criteria | |
| --- | --- |
| 1 | Articles in English, or those having detailed summary in English. |
| 2 | Studies published between 1^st^ January 1981 and 31^st^ December 2011. |
| 3 | Primary research and cross-sectional data at community level with essential data for calculating prevalence. |
| 4 | Study population was apparently healthy and was a tribe (tribe as deemed by study authors or as mentioned in Scheduled Tribe list of Article 342 of the Constitution of India). |
| 5 | Studies that provided information for age groups ≥18 years and either sex. |
| 6 | Well defined diagnostic criteria for hypertension. |
| 1. Exclusion criteria | |
| 1 | Study population was exclusively < 18 years or > 60 years. |
| 2 | Studies that provided inadequate information for calculating prevalence. |
| 3 | Studies that reported pre-diagnosed or self-reported hypertension. |
